# Advancing solid-state fermentation with culinary fungi for nutrient-dense, minimally processed, whole-foods and resilient food systems—a narrative review

**DOI:** 10.3389/fnut.2026.1798618

**Published:** 2026-04-15

**Authors:** Suzannah Gerber, Scott C. Frost, Alexis M. Walker, David L. Kaplan

**Affiliations:** 1 Center for Cellular Agriculture, Tufts University, Medford, MA, United States; 2 Division of Agriculture, Food and Environment, Gerald J. and Dorothy R. Friedman School of Nutrition Science and Policy, Tufts University, Boston, MA, United States; 3 Department of Food and Nutrition Studies, George Mason University, Arlington, TX, United States; 4 Auster Center, Gordon Institute, School of Engineering, Tufts University, Medford, MA, United States; 5 Department of Biology, School of Arts and Sciences, Tufts University, Medford, MA, United States; 6 Department of Biomedical Engineering, School of Engineering, Tufts University, Medford, MA, United States

**Keywords:** agricultural valorization, food security, mushrooms, mycelium, nutrient-density, protein bioavailability, sensory and flavor development, solid-state fermentation

## Abstract

**Introduction:**

Solid-state fermentation with culinary fungi (mushrooms commonly used in cooking) could be a scalable means of producing nutrient-dense protein foods, while improving digestibility and bioavailability and valorizing agricultural commodities and byproducts. Advancing the science of these processes has major implications for both nutrition security and resilient, sustainable food systems. This review synthesizes how substrate selection, fungal strain choice, and controllable growth conditions can transform underutilized or under-consumed plant materials into minimally processed, consumer-acceptable foods through enhanced sensory characteristics and nutritional profiles.

**Methods:**

We conducted a narrative review to synthesize peer-reviewed and applied literature to map substrate–strain–process combinations that could enhance nutritional composition, sensory performance, food safety, scalability, and sustainable food-system outcomes.

**Results:**

Across cereals, legumes, and oilseed meals, solid-state fermentation consistently increases protein concentration and quality, improves nutrient bioavailability, decreases anti-nutritional factors, and generates flavor-active metabolites while enhancing texture. Outcomes can be further optimized through manipulation of growth conditions such as moisture, temperature, particle size, aeration, light, residence time, and post-process thermal finishing.

**Discussion:**

Integrating culinary fungi with commodity crops enables circular use of side streams, shortens protein production cycles relative to animal sources, and can support rural economies while aligning with scientific dietary guidance. Factorial process studies linking growth stage inputs to sensory acceptance, standardized safety and regulatory frameworks and techno-economic analyses that quantify cost-per-nutrient and edible output per hectare would help extend this knowledge for maximal impact.

**Conclusion:**

Solid-state fermentation should be considered as an important strategy for improving diet quality and food security.

## Introduction

Solid-state fermentation (SSF) is a bioprocess in which microorganisms, typically fungi, colonize solid substrates. SSF has emerged as a practical approach to transform edible base materials into nutrient-dense foods. Compared to other fermentation techniques, such as submerged liquid-state fermentation (LSF), SSF has received less academic and industry attention (1, 2). SSF and LSF are different but complementary platforms. LSF produces a more homogeneous, tightly controlled, concentrated mycelial biomass grown in liquid media, whereas SSF is a low-water transformation of a solid substrate into a composite, minimally processed food that includes the mycelium itself. Recent research highlights the capacity of SSF to improve protein quality by concentrating essential amino acids and enhancing digestibility of otherwise recalcitrant biomass (3), while generating savory flavor precursors under growth conditions that require relatively low water and energy inputs (1, 2, 4–7). Importantly, these benefits extend beyond fungal protein concentration alone.

Many alternative protein production systems depend on extraction and intensive processing. By contrast, fungal mycelia grown on edible substrates enable integrated, minimally processed composites that preserve the nutritional and sensory properties of whole food (e.g., whole grains, legumes, and seeds). In addition, the use of commodity crops and their side streams as SSF substrates provides an avenue for additional valorization, resulting in significant nutritional, sensory, and economic opportunities for SSF production (5, 8). SSF could be an important lever to support alignment of dietary patterns with public health guidance, increase the consumption of whole and minimally-processed foods, reduce on-farm and food waste, add value to commodity crops, and increase access to protein-rich, plant foods (5). Traditional exemplars of SSF fermentation such as tempeh (e.g., *Rhizopus* SSF of soybeans) and oncom (e.g., *Neurospora* or *Rhizopus* SSF of peanut or soy oil press cakes) have converted agricultural products and side streams into high-protein foods with greater digestibility for hundreds of years (3). However, these foods are primarily eaten in East Asia and adoption elsewhere is low, in part due to hedonic rejection. In contrast, modern food manufacturers have recently introduced novel mycelium-forward products (e.g., Quorn™, Meati™, Prime Roots™) that have gained traction in Western markets. Recent articles and reviews largely frame SSF as a tool to produce meat analogs, or further examine methods of enhancing traditional tempeh or oncom (3, 9, 10) reflecting that mycelium visibility has been driven by submerged fermentation, while SSF is emerging as a complementary solid-state platform (11). Consequently, relatively few analyses consider how SSF can serve as a standalone method for producing sensory-optimized, minimally processed, nutrient-dense foods on inherently nutritious edible substrates. Even fewer examine how coordinated choices across substrate, strain, and growth-condition vectors could enable broader global adoption than traditional SSF foods. Such synthesis of information on combinations can be applied to expand product development and also open up additional market opportunities for crops and edible sidestreams (5, 12).

**Figure 1:**
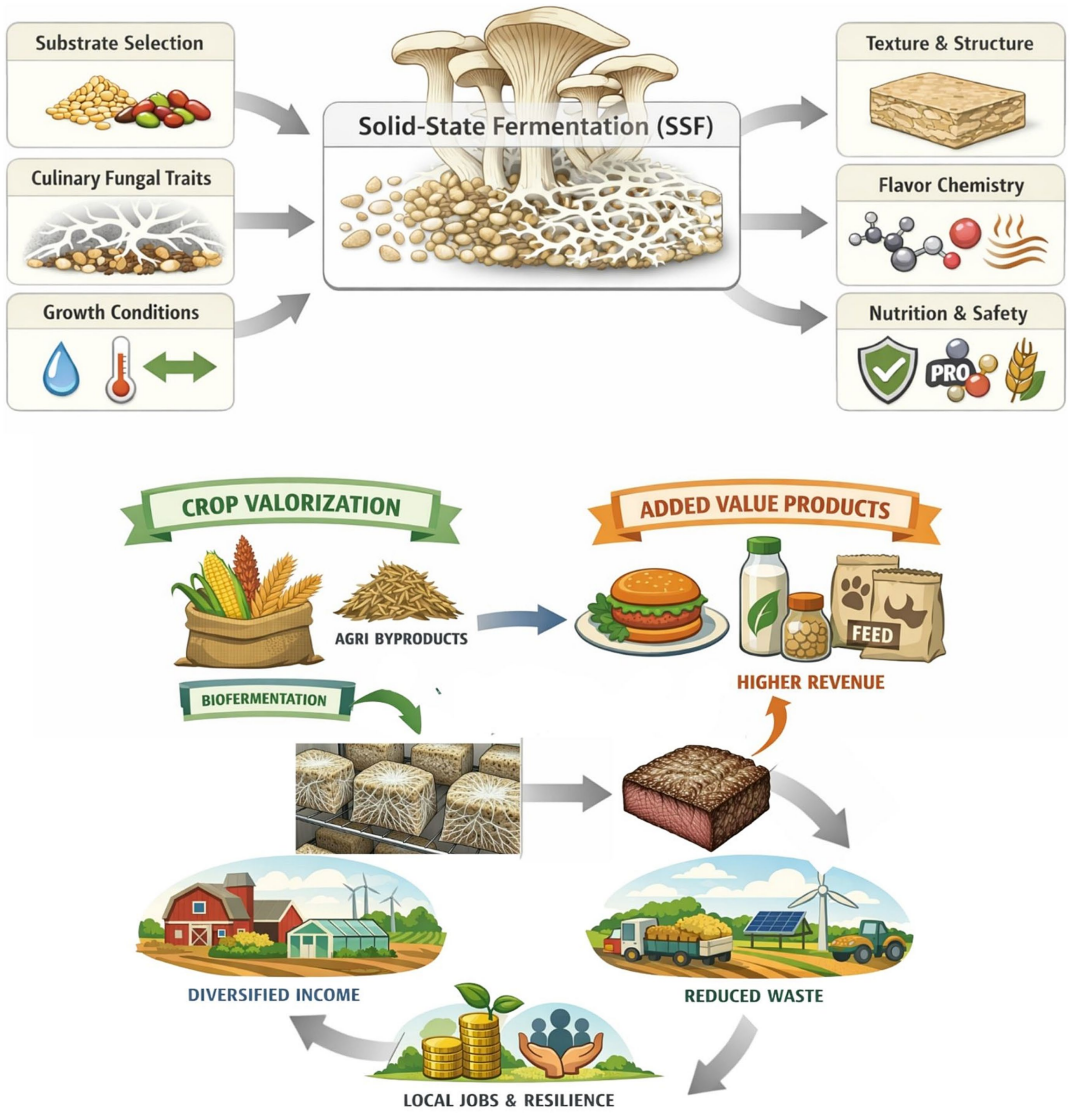
Conceptual pathway for whole-food solid-state fermentation with culinary fungi and food-system impacts.

Despite nutritional and sustainability advantages, SSF remains less commercially utilized relative to LSF. This may be due to various tunable factors such as heat/mass-transfer, limited in-line monitoring, and substrate features that are standardized across processes used in LSF (1, 13). This review synthesizes practical solutions to mitigate the challenges of SSF and outlines a targeted research agenda to accelerate reliable, food-grade deployment, while summarizing the known nutritional and sensory results of SSF connected to different inputs and growth conditions. The information that follows can help outline key advancement steps for stakeholders—from scientists, product developers, regulators, to agricultural producers and others—working to create more nutrient dense, minimally processed foods. The following sections outline optimization pathways, with attention to edible substrate combinations and the use of culinary mushroom strains (mushrooms and edible fungi valued for their taste, texture, and use in cooking) to enhance final food matrices. This review complements recent SSF literature focused on “meat analogs,” (5) incorporating broader considerations of substrate composition, food-system impacts, commercial progress, and safety and regulatory frameworks. We synthesize quantitative nutrition effects—including protein concentration, accrual, and reductions in anti-nutrients—and relate these biochemical changes to nutrient availability and sensory attributes. Additional technoeconomic hotspots, such as moisture, particle size, aeration, and temperature influences on growth time and yield are identified as key parameters for improving unit cost and consumer willingness to try and buy. Finally, we situate SSF within global nutrition-security and circular-bioeconomy agendas, highlighting its capacity to upgrade crop value, diversify farm income, and provide minimally processed, nutrient-dense foods to broad populations (Figure 1).

This narrative review focuses on culinary fungi with established or emerging relevance to SSF. We began with traditional models (e.g., tempeh, oncom) and commercially developed mycelium products (e.g., LSF, such as Quorn), then searched for papers describing SSF using varying substrates to compare reported yield, nutritional, sensory, and resource-use outcomes. Using sentinel papers (1, 10, 11, 14–17) and common culinary fungi we conducted targeted searches in PubMed and backward/forward citation searches in Web of Science for each fungal strain and application area, using keywords relevant to sentinel papers and additional search terms relevant to substrate, crop valorization, nutrient density and bioavailability, safety, sensory and texture outcomes, sustainability, and regulatory considerations. We included studies on food-grade, minimally processed SSF using edible fungi, along with relevant liquid-state comparators. As a narrative review, we aimed to include all identified relevant papers rather than apply predefined exclusion criteria or conduct a comprehensive systematic search.

## Advantages for using commonly consumed culinary fungi for SSF

Most commercially available LSF and SSF use fungal strains (e.g., *Fusarium. venenatum*, and *Rhizopus oligosporus*) with no direct culinary applications beyond use in mycelium-based foods or to produce mushroom fruiting bodies for human foods. However, mushroom fruiting bodies have many culinary uses, lauded for their firm, meaty texture and savory flavors (18). For edible substrate SSF, choice of fungal species can intentionally alter the taste and texture of the final output, and be used to capitalize on the sensory advantages of popular mushrooms. The most relevant fungal traits for SSF include: (i) colonization kinetics and growth robustness on food-grade substrates; (ii) extracellular enzymes (e.g., amylases, cellulases/hemicellulases, pectinases, proteases, phytases) able to metabolize a range of plant materials; (iii) mycelial packing density and hyphal morphology (i.e., rhizomorph tendency, branching) and (iv) resultant production of sensory relevant metabolites (e.g., nucleotides, free glutamate/peptides, lipids, and volatiles) (19–24). Culinary fungi species selection should complement growth substrate to achieve optimal texture, flavor and nutritional outcomes. Below we highlight popular strains and their known benefits and drawbacks for SSF.

### *Pleurotus* spp. (oyster mushrooms: *Pleurotus. eryngii*, *Pleurotus. ostreatus*, *Pleurotus pulmonarius*)

Among culinary mushrooms, *Pleurotus ssp*. consistently demonstrate strong performance on edible substrates including the ability to rapidly colonize on hydrated grains, legumes and fibrous co-substrates; secrete robust carbohydrases and proteases to metabolize otherwise indigestible fibers such as lignocellulosic wastes; liberating amino acids for enhanced protein digestibility (25); form dense, cohesive mycelial networks that bind particles into sliceable slabs for meat analogs; or milled into flour with increased moisture retention in baked goods (21, 26, 27).

SSF studies with *Pleurotus ssp.* on canola and cottonseed meals, or cereal–legume bases, report increased protein, reduction in anti-nutrient factors (e.g., phytate, sinapine, gossypol, glucosinolates, purines), and gains in antioxidants and polyphenols (28–37). During *Pleurotus ssp*. growth, secreted enzymes degrade structural polysaccharides, improving substrate digestibility for humans (38). Together, rapid colonization, broad extracellular enzyme portfolios, and dense, cohesive mycelial networks, make *Pleurotus ssp.* a pragmatic genus for SSF, and one capable of converting agricultural side streams and whole crop substrates into nutrient-dense, sensory-forward, value-added foods.

Among individual species*, Pleurotus eryngii* (i.e., king oyster mushroom, or King Trumpet Mushroom) may be the strongest species within the genus, with robust rhizomorphic growth, dense mycelial mats, and tolerance for modest lipid inclusion (19, 25, 39). Studies of *P. eryngii* are able to valorize low-acceptance, high yield, and resilient crops by increasing consumer acceptance of the resulting foods (28, 40, 41). One study of SSF of canola meal with *P. eryngii* found greatly enhanced nutritional profile with an 11–18% increase in protein content and improved *in vitro* digestibility when fermented for 12 days (12). *P. eryngii* is especially attractive for it is ability to produce a flavorful, dense steak or other whole meat cut format consumed as a single whole mycelium-substrate (5). Lastly, because both *Aspergillus* and *Rhizopus* include oleaginous species that enhance flavor profiles through lipid accumulation and enzymatic generation of flavor-active compounds, co-culturing *P. eryngii* with *Rhizopus* or *Aspergillus* could help to widen substrate utilization and tailor for specific flavor/texture outcomes (42, 43).

### Lentinula edodes (shiitake)

*Lentinula* fruiting bodies are known for producing elevated levels of guanylic acid (i.e., Guanosine 5′-monophosphate, or 5′-GMP), free glutamate, and sulfur-derived volatiles (e.g., lanthionine) that drive savory flavor. Additionally, *Lentinula* mushroom is known for its cap with a firm, meat-like texture, and distinctive aroma profile. However, *Lentinula* requires lengthy colonizing time to achieve fruiting (44, 45). *Lentinula* mycelium can produce cohesive networks but is less rhizomorphic than *P. eryngii*, and the resulting mats are less dense, making it more suited to minces or used as flavorful additive where *Lentinula* may be valuable as a flavor driver contributing umami-active nucleotides rather than standalone foods or whole cut meat analogs. However, there is great opportunity to explore hybrid co-cultures (such as with rapidly colonizing *Rhizopus*) to add a favorable aroma while accelerating colonization and forming denser mycelial mats (19).

### Agaricus bisporus (button/portobello)

*Agaricus* is a ubiquitous culinary mushroom, valued for its plentiful growth and mild flavor. However, its mycelium typically forms a delicate network that lacks the packing density required to generate cohesive, whole-cut structures. Although *Agaricus* has a balanced essential amino acid profile, it generally has lower concentrations of flavor-active compounds compared to other culinary fungi making it less appealing for some meat analogs (44, 45). However, use of the fruiting body of *Agaricus* can be a reliable co-culture partner with structure and flavor supplied by *Pleurotus* or another companion fungi.

### Hericium erinaceus (lion’s mane)

*Hericium* has gained increased popularity in culinary applications, largely due to its fibrous, fruiting bodies which have been explored in the production of seafood alternatives, especially shellfish such as crab and shrimp (46). Its mycelium, which is typically cottony and slower to colonize, often fails to form dense substrate mats, but still contributes distinctive flavor and texture attributes from pinning through fruiting. Fruiting bodies have multiple applications from shredded or “pulled” meat applications, owing to lighter and fibrous textures, which make *Hericium* fruit valuable not only for seafood applications but also as a broader flavor adjunct (19, 44, 45). In addition to culinary use, *Hericium* has attracted attention for its bioactive compounds and potential applications in functional foods and nutraceuticals, which could be leveraged to increase interest for SSF foods (47–49).

### Other culinary fungi—co-culture candidates

Additional culinary basidiomycetes offer potential for SSF applications, though their growth characteristics and technoeconomic profiles differ from the aggressive colonizers described above (50). *Flammulina velutipes* (enoki) and *Hypsizygus marmoreus* (beech) are widely consumed mushrooms with mild flavors and light coloration which can be advantageous for neutral-tasting matrices. Additionally, both species produce fine, sparsely rhizomorphic hyphae and slower substrate colonization than other *Pleurotus s*sp. (19, 44, 45). *Flammulina* can contribute subtle umami notes, polysaccharide enrichment, and textural softening (51), while the mild aromatic compounds of *Hypsizygus* could be used to modulate flavor, offering attractive traits for innovative co-culturing.

*Auricularia* sp. (wood ear) are a common culinary mushroom with a distinct gelatinous texture, and polysaccharide-rich matrix high in soluble fiber and *β*-glucans. These features offer unique opportunities for moisture retention, chewiness, and functional fiber enrichment in SSF composites (52–54). Studies of *Auricularia* spp. describe moderate mycelial growth rates highly dependent on substrate composition and culture conditions, with colony expansion and mycelial density optimized within a narrow carbon–nitrogen range (55, 56). Additionally, *Auricularia* ssp. provides moderate mat cohesion (56) which makes *Auricularia* beneficial for SSF in co-culture systems where its mucilaginous texture can complement a structurally robust companion strain (55).

*Grifola frondosa* (maitake) is valued for its rich umami profile, aromatic complexity, and notably high *β*-glucan content especially the *β*-1,3- and *β*-1,6-glucans in mycelium (57). *Grifola* polysaccharides form branched glucan networks, yielding mycelium that is cohesive but not densely rhizomorphic. As a result, *Grifola* typically exhibits only moderate structural integrity and slower substrate colonization than more aggressive genera such as *Pleurotus*. However, its robust metabolic activity produces many functional bioactive metabolites which help give maitake its well-known flavor profile, making it attractive for both flavor development and nutritional enhancement (58). However, because its mycelial architecture lacks the dense binding needed for strong matrix formation, *Grifola* may also perform best as a co-culture, to leverage sensory and nutritional qualities without relying on it for rapid substrate conquest.

## Nutritional profile of mycelium-rich foods and SSF benefits

Edible mushroom mycelium is a nutritionally rich food source often compared to animal proteins (5, 11), with both mycelium and fruiting bodies high in protein (20–30% of dry weight, and some as high as 40%, depending on growth conditions) (38) with a balanced essential amino acid profile (59). For example, the mycoprotein produced by *Fusarium venenatum* (e.g., Quorn™ foods) has a protein digestibility-corrected amino acid score (PDCAAS) of ~0.996^44^, which can be comparable to chicken and beef, but is much higher than soybeans (60–63). Many fungi are also “complete” proteins (having a balance of all essential amino acids) but also have high levels of functional amino acids associated with flavor and animal-sourced foods (e.g., glutamate, GABA) (38). Alongside protein enrichment from the fungal biomass accretion itself, reproducible nutritional benefits of SSF include (i) liberation of bound polyphenols and consequent gains in antioxidant capacity (64–66); (ii) reductions in anti-nutritional factors, especially phytic acid in cereals or legumes and glucosinolates and sinapine in Brassica meals which increases nutrient availability (67); (iii) formation of flavor-active molecules (free amino acids, short peptides, and 5′-nucleotides) that increase umami perception; (iv) increases in dietary fiber (especially polysaccharides like *β*-glucans found in fungal cell walls), which have been associated with cholesterol-lowering and immune-modulating effects (11, 38, 68, 69); and (v) increases in essential nutrients such as B-vitamins, ergosterol (provitamin D2) (70, 71), zinc, potassium, copper, and selenium. In contrast to other sources of these nutrients (plant and animal), the lipid content, and consequently the relative energy density, of mycelium is low, resulting in health-promoting, nutrient dense foods (38, 60).

Two complementary mechanisms drive nutritional gains in SSF (Table 1). First, the fungus contributes its own mycelial biomass, producing high quality protein, fiber (typically 20–35% dry basis in mushroom mycelium), *β*-glucans and chitin (72–74), B-vitamins [notably riboflavin, folate, and when co-cultured or fortified vitamin B12 (5)], and ergosterol (provitamin D2) which can be further modulated by UV exposure to stimulate greater endogenous production (71). As mycelium preferentially metabolize carbohydrates to support growth, the relative protein content of the substrate increases, and concurrent mycelial activity releases peptides and amino acids that contribute to improved protein digestibility (5, 12, 38, 75, 76).

**Table 1:** Summary of outcomes from edible-fungi solid-state fermentation (SSF) applied to diverse food substrates.

Outcome target	Representative SSF system(s)	Quantitative effect examples	Notes / Implications	Key sources (year)
Protein content and bioavailability Increases	Canola meal × *Pleurotus ostreatus* (≈12 d); Soybeans × *P. ostreatus* (long run); Peanut press cake (oncom) × *Neurospora sitophila* / *Rhizopus oligosporus; Okara × R. oligosporus / A. oryzae; Tempeh soy × Rhizopus*	Content: +11–18% (canola); +27–28% (soy, 31 d); 52.6–55.35% (peanut press cake, dry basis); Essential AAs of ≥ 12.06 g/100 g; Digestibility increased quality; >50% of soy protein became free amino acids (tempeh)	Mix of concentration effect + *de novo* fungal protein; enables protein-dense composite foods. Fungal amino acid spectrum complements legumes/cereals Protease/peptidase action.	(3, 28, 42, 73, 86, 89, 140, 184, 208, 209)
β-glucan Increases	Cottonseed cake + *Lathyrus* (80:20) × *P. ostreatus* (11 d); *Fusarium venenatum* mycoprotein and binding of bile salts	5 × increase in Beta-glucan content; Mycoprotein fiber 24% (DW), 2/3 β-glucans and 1/3 chitin	Adds soluble fiber with immune-metabolic potential (131); texture contribution. *In vitro* digestion of mycoprotein reduced lipolysis and bound bile salts, a mechanism associated with reduced blood cholesterol in humans	(25, 29, 69)
Reduced Anti-Nutrients (phytates, glucosinolates, sinapine, gossypol)	Canola meal × *P. ostreatus*; Oats/Barley × *Rhizopus* (tempeh-style); Cottonseed + Lathyrus × P. ostreatus	Phytates: −55–76% (canola); oats −74%, barley −89%; Glucosinolates: −98.8%; Sinapine: 99.8%; Gossypol: total reduction 60–80%, with some reporting up to −89%, and free gossypol −12.45%	Increases Fe/Zn accessibility; supports bioavailability gains; Major removal of pungent/bitterness drivers; flavor improvement; increased protein digestibility	(9, 10, 28, 29, 36, 41, 106, 115, 143)
Aflatoxin Reduction (contaminated inputs)	Peanut press cake (black oncom) × *Neurospora* / *Rhizopus*	−50–70%	Requires tight process control and clean sourcing	(3)
Increased antioxidants and phenolics	Soy × *P. ostreatus* (long SSF); Okara × *R. oligosporus* / *A. oryzae*; Oats × *Rhizopus; Oats × R. oryzae; Okara × Rh. oligosporus / A*sp. *oryzae; Soybeans × P. ostreatus*	Soy phenolics 4.47×, DPPH 3.92×; Okara +260–550% antioxidant; Oats total phenolics increase	Liberation of bound phenolics; flavor co-benefits of reduced bitterness and astringency; longer shelf-stability	(10, 42, 64–66, 115, 140)
Omega fatty acids (ω-3) profile	Grass pea + flax press cake × *Rhizopus* (tempeh)	ALA increases by >10×; ratio of ω6:ω3 from 11:1 to 0.5–2.5:1	Co-substrate strategy; retains PUFA while carbs fuel growth	(10, 99)
Vitamin & Mineral content and absorption increases	Okara (red oncom) × *Neurospora intermedia; Barley meal × Rhizopus (tempeh-style) vs boiled barley*	Ca 215 mg/100 g; P 66 mg/100 g; Fe 12.5 mg/100 g; non-heme Fe absorption 5.5% vs. 3.0% (+83% relative); Vitamin B1 raised to 150 μg/100 g	Demonstrates micronutrient density in fermented matrices	(van Veen & Sohaefer 1950) (3, 9, 10, 32, 80, 89)
Flavor balance improvements	Barley/oats × *Rhizopus* (with/without yeasts); SSF soy/cereal × Pleurotus/Rhizopus; Brassica meals × Pleurotus; cereal/legume tempeh × Rhizopus	Reduced grassy, beany flavor ratings; increased glutamate, 5′-GMP/IM savory flavor volatiles; increased pyrazines, Strecker aldehydes, 2-acetylpyrrole for cooking step flavor expression; Sensory bitterness falls as sinapine/IP6 decline	Sensory lift via proteolysis + ribonucleotides + Maillard on cooking	(15, 19, 28, 41, 42, 44, 106–108, 111, 142)
Texture, moisture and cohesion improvements	Cereal/legume SSF flours; Pleurotus (esp. *P. eryngii*)	higher water holding capacity (WHC); better juice release; elastic, sliceable networks (hyphal entanglement); Dense, anisotropic mycelial mats; Moisture 55–65%; 2–4 mm particle size; O₂ diffusion	Enables anisotropic, meat-like bite with minimal texturizing	(19, 25, 42, 110, 116)
Allergenicity	Buckwheat soba (tempeh-processing) × *Rhizopus*; (lupin proteomics in SSF, various); Lupin × Rhizopus; peanut press cake oncom × Neurospora/Rhizopus	Loss of allergen bands; reductions in IgE not always reported; Lupin: β-conglutin peptides reduce; Peanut: IgE binding slightly lower.	Promising but awaiting more research to be clinically validated	(10, 101, 106, 208)

The table compiles representative substrate × microorganism systems and the typical quantitative effects reported for key endpoints. Values reflect magnitudes reported in the cited studies and will vary with strain, inoculum and distribution, substrate composition/particle size, moisture/water activity, temperature, aeration/bed design, and process time; rows should therefore be interpreted as evidence exemplars rather than directly comparable benchmarks. Amino acids (AAs); essential amino acids (EAA); β-glucans, 1,3/1,6-β-D-glucans; 2,2-diphenyl-1-picrylhydrazyl radical-scavenging (DPPH) assay; guanosine/inosine 5′-monophosphate 9 (5′-GMP/5′-IMP,); polyunsaturated fatty acids (PUFA); α-linolenic acid (ALA); omega-6:omega-3 ratio (ω-6:ω-3); phytic acid (myo-inositol hexakisphosphate; IP6).

The second mechanism is driven by secreted enzymes that biotransform the substrate. Many plant foods (e.g., legumes, wheat bran, spinach) contain “anti-nutrients” such as phytic acid, and tannins (Table 2) which bind essential minerals (i.e., iron, calcium) thus reducing their bioavailability (77). However, the enzymatic activity of fungi can degrade these molecules, leading to improved nutrient absorption (5, 78–81). For example, fermentation of rapeseed (canola) meal by *Pleurotus* mycelium degrades sinapine and phytates, while concentrating protein (35, 82), and was similar for *Pleurotus* fermentation of mixed grain or legumes (5, 29, 83). Carbohydrases, proteases, and phytases improve protein digestibility by hydrolyzing macromolecules to release bound phenols, flavonoids, and peptides, and by degrading anti-nutritional factors including phytic acid (an iron- and zinc-chelating inhibitor of protein digestibility), sinapine and glucosinolates (bitter/pungent compounds), and gossypol (a toxic cottonseed phenolic) (32, 33, 73, 74, 84–86). Additionally, many industrial mycoprotein processes include an RNA reduction heat step to neutralize purines (87, 88). Together, these increase nutrient availability from common plant food bases and the mycelium itself (89). Results can be tuned through substrate pretreatment (hydration, gelatinization, altered particle size), moisture (60–65%), temperature (24–30 °C for *Pleurotus*), and aeration, which modulate colonization by influencing heat removal, gas exchange, water activity, permeability, hyphal ingress, and enzyme secretion (43, 90–96).

**Table 2:** Anti-nutrients and potentially harmful compounds in SSF feedstocks and fungal biomass, and mitigation pathways enabled by solid-state fermentation (SSF).

Compound	Foodstuff source examples	Effects on consumption	Example impacts of combining through SSF	References
Phytate	Brassica meals, cereals, okara, brans, spent grains, pomace, pseudocereals	Chelates Fe, Zn, Ca, Mg, lowering bioavailability	Fungal phytases (e.g., Rhizopus, Aspergillus, Pleurotus) produce phytases that hydrolyze phytate releasing bound minerals	(9, 22–24, 28, 41, 98, 115, 144)
Glucosinolates	Brassica meals (canola, mustard), cruciferous vegetables	Goitrogenic effects, thyroid dysfunction, bitter taste, reduced animal performance	Fermentation with Rhizopus oligosporus, Lactobacillus spp., or Bacillus spp. to hydrolyze glucosinolates into less harmful compounds	(28, 32, 35, 211)
Sinapine	Brassicas (Rapeseed, mustard seed, and others)	Bitter, astringent taste; reduced protein digestion; may cause allergic response	Solid-state fermentation with Trametes sp., Rhizopus oligosporus, or *Bacillus subtilis* degrades sinapine via laccase and other enzymes	(10, 23, 28, 32, 36)
Bound phenolics	Legumes, cereals (wheat), rapeseed, canola, quinoa, fruits, vegetables	Reduced protein/mineral bioavailability, astringency, reduced digestibility	Fermentation with Rhizopus oligosporus, *Aspergillus niger*, Pleurotus ostreatus, or *Lactobacillus plantarum* to release bound phenolics	(10, 33, 66, 85, 89, 115, 140, 141)
Tannins	Legumes, cereals, nuts, tea, fruits, vegetables	Reduced protein digestibility, astringency, toxicity at high intake	Fermentation with tannase-producing fungi (e.g., Penicillium glabrum, Aspergillus glaucus, *A. niger*, Rhizopus sppand others) to hydrolyze tannins	(Traka, 2016) (50, 78, 79, 113, 114)
Protease inhibitors/lectins	Legumes (soybeans, kidney beans, chickpeas), cereals	Inhibit digestive enzymes, reduce protein utilization, cause pancreatic hypertrophy	Fermentation with Rhizopus oligosporus, Aspergillus oryzae, *Bacillus subtilis*, or protease-secreting fungi to degrade inhibitors/lectins	(32, 50, 62, 63, 67, 77, 141, 143)
Allergenic proteins (β-conglutin)	Lupin seeds (*Lupinus angustifolius*, *L. mutabilis*), fungal/yeast biomass	IgE binding in sensitized individuals and allergenic reactions, immune hypersensitivity, reduce nutrient intake through increased excretion	Proteolysis during SSF reduces specific peptides; Fermentation with Rhizopus oligosporus, Propionibacterium spp., or protease-producing fungi to hydrolyze allergenic proteins (should be validated with immunoassays)	(3, 101–103)
Gossypol	Cottonseed meal or cake	Toxicity (liver, reproductive, cardiac), binds lysine	Solid-state fermentation with *Candida tropicalis*, *Saccharomyces cerevisiae*, *Aspergillus niger*, Pleurotus spp., or Paecilomyces variotii	(29, 32, 37, 84)
Saponins	Legumes (soybeans, chickpeas), quinoa, green microalgae	Bitter taste, hemolytic activity, reduced nutrient absorption	Fermentation with Rhizopus oligosporus, Aspergillus spp., *Saccharomyces cerevisiae*, or lactic acid bacteria to degrade saponins. SSF can alter saponin profile (sometimes reducing, sometimes increasing saponins); pre-washing/dehulling and post-processing are often required for reliable debittering.	(50, 210, 211
Purines (nucleic acids)	Yeast-rich biomass, fungi, legumes	Uric acid load (hyperuricemia) for gout	Fermentation with Aspergillus oryzae, Blastobotrys adeninivorans, *Candida utilis*, or low-purine yeast strains; enzymatic degradation. Downstream RNA reduction is described by commercial operators using heat-shock to activate endogenous RNases as a standard mitigation step for fungal biomass intended for high intake.	(32, 34)
Chitin/Beta-glucans	Fungal biomass, mushrooms, yeasts, microalgae	Reduces apparent protein digestibility in vitro; Associated with indigestion; tough chewing texture; potential allergenicity	Fermentation with chitinase/β-glucanase-producing fungi (Mucor rouxii, *Aspergillus* terreus, Trichoderma spp.). Milling, alkaline/thermal pretreatments, or targeted enzymatic hydrolysis can further break down to improve digestion.	(58, 68, 73, 74, 109)
Mycotoxins	Contaminated grains, nuts, by products	Toxicity (carcinogenicity, nephrotoxicity, immunosuppression, reproductive toxicity); carcinogens, hepatotoxicity, immunosuppression	Fermentation with mycotoxin-degrading fungi (*Rhizopus* spp., a-toxigenic strains of *Aspergillus* spp.); laccase/peroxidase enzymatic detoxification (lactonases, peroxidases, laccases); Aflatoxin-degrading fungi (*Trichoderma reesei*, *Aspergillus niger, Rhizopus* spp.)	(3, 32, 195, 199)

Effects of SSF are substrate-, strain-, and process-dependent. In some matrices, reductions in anti-nutritional factors or increased phytase activity do not necessarily translate into improved in vitro digestibility (143), and quinoa saponins may increase or decrease depending on processing conditions (Gautheron et al., 2024). Validate key endpoints analytically for the target substrate and product format.

Substrate composition can also impact product nutrition (Tables 1, 2). In SSF of brassica meals (e.g., canola, rapeseed), *Pleurotus ostreatus* increased protein content (11–18% dry weight), but reduced sinapine (99.8%), glucosinolates (~98.8%), and phytate content (~56–76) (28). Studies using cottonseed press cake blended with *Lathyrus* (i.e., sweet peas) pericarp (80%/20%), *P. ostreatus* reported increased crude protein content (35%), and 1,3/1,6-*β*-glucans were five times greater, but total gossypol was decreased (89%). These addressed a key safety and sensory constraint of cottonseed substrates, where the presence of gossypol prevents safe consumption for humans and palatability; mycelial fermentation is a promising alternative to genetic modification of cottonseed, the only other viable approved path for human consumption (29, 97). Comparable outcomes have been observed for *P. ostreatus* on soybeans, resulting in elevated protein without isolation and increased antioxidant levels (42).

In cereals (e.g., oats, barley) fermented with *Rhizopus oligosporus* like tempeh, phytate content dropped by 74 and 89%, respectively. In a human crossover study, iron (non-heme iron) absorption from fermented whole-grain barley reached 5.5% versus 3.0% for boiled barley, demonstrating a bioavailability benefit consistent with phytate reduction (41, 98). Co-fermenting grass pea with flaxseed oil-cake strategically re-profiles lipids: *α*-linolenic acid (ALA, *ω*-3) rose 3.6–10 × (dose-dependent) and the ω6:ω3 ratio improved from 11:1 to 0.5–2.5:1, with modest increases in sulfur amino acids—an approach that leverages lipid-rich inputs while the fungus primarily utilizes carbohydrate (99).

SSF can also reduce allergenicity of certain foods (Table 1). Fermentation breaks down large proteins into smaller peptides and amino acids, degrading many IgE-binding epitopes of foods like soy, lupins, wheat, and peanuts (100, 101). For example, fermenting peanut flour with *Rhizopus* for 1–2 days significantly degraded peanut allergen proteins (102, 103).

SSF thus offers a pragmatic route to valorize crops and edible side streams while enhancing nutritional value (67). Below, we add additional insight on sensory enhancement and circular-economy, to show how SSF with edible fungi addresses complementary priorities for nutrition security and sustainable food systems.

## Sensory and consumer experience advantages for SSF

Mushroom aroma is often attributed to C₈ compounds such as 1-octen-3-ol and 1-octen-3-one, but the volatile profile of mushrooms is far more complex, including a broader ensemble of lipid-derived alcohols, aldehydes, ketones, and sulfur (104). As fungi colonize a substrate, their metabolism will reflect the consumption and modification of substrate derived nutrients creating, diminishing, or amplifying key sensory attributes. Secreted fungal enzymes can further modify substrate chemistry, generating new volatile and non-volatile compounds that contribute to aroma, taste, and texture. These can be influenced by moisture levels and particle sizes which support oxygen diffusion and consistent colonization, as well as controlled post-fermentation through thermal processing to amplify Maillard/Strecker pathways (15, 105, 106).

SSF flavor development is strongly influenced by proteolysis, ribonucleotide metabolism, and substrate-specific biochemical pathways. These processes can increase the abundance of taste-active compounds such as L-glutamate, guanosine 5′-monophosphate, and inosine 5′-monophosphate, associated with umami flavors of culinary mushrooms and traditional SSF like tempeh (19, 44, 45, 107, 108). Furthermore, studies of *Pleurotus* spp. have also demonstrated enhanced umami intensity through enzymatic activity and nutrient turnover (109). Additionally, SSF can influence the development of thermally driven aromatics by building the precursor pool of reducing sugars, free amino acids, and peptides essential for Maillard and Strecker reactions during cooking. As a result, thermal finishing (e.g., baking, roasting, pan-searing) of fermented substrates often yields elevated Strecker aldehydes (e.g., methional, phenylacetaldehyde), alkylpyrazines (e.g., 2-methyl-, 2,5-dimethylpyrazine), and heterocycles such as 2-acetylpyrrole, contributing roasted and nutty aromas (15, 106, 110, 111). Studies evaluating SSF-treated vs. mushroom-enriched baked foods found increased intensities of nutty, roasted, and mushroom-like aroma, along with umami and sweet taste modifications. Further, consumer evaluations showed that acceptance of fermented products was modulated by moisture content and fiber level, enhanced in fermented mixes (106, 110, 112).

SSF can also remove odor-active precursors (Tables 1, 2). Across grains, legumes, and oilseed press-cakes, SSF consistently reduces bitterness and astringency through the degradation of phytate, tannins, and sinapine (77, 113–115). In Brassica meals, observed reductions in sinapine and glucosinolates by *Pleurotus* effectively lower the pungent mustard-like notes and bitterness (27, 82). Ferments using *Rhizopus* with or without lactic acid bacteria amendments can be used to suppress beany and grassy aromas while imparting mild acidity. SSF fungi can also increase umami flavor and roasted aromas through accrual of amino acids and reactant sugars, both Maillard chemistry precursors.

However, SSF does carry risks of negative sensory expression which must be controlled during growth. Under nutrient imbalance or suboptimal moisture conditions, fungi may produce elevated levels of volatile sulfur compounds, geosmin-like earthy notes, or lipid-derived aldehydes associated with rancidity (105, 116–119). Overly aggressive enzymatic activity may also weaken substrate structure, resulting in soft, crumbly, or overly fibrous textures that can detract from product quality (2, 110). SSF can be strategically tuned to enhance desirable flavors, suppress off-notes, and selectively reshape challenging substrates.

In addition to modulating aroma and taste through metabolic and biochemical pathways, SSF also alters the physical structure of the substrate. These structural effects play a central role in defining the texture of the final product. With continued growth, enzymatic hydrolysis of structural carbohydrates and the expansion of hyphal networks jointly modify substrate mechanics which impact sensory enjoyment and culinary functionality. Two primary mechanisms dominate texture formation during SSF. The first mechanism is the partial enzymatic hydrolysis of abundant structural polysaccharides within the substrate (e.g., hemicellulose and pectin). As these polysaccharides are broken down, the matrix becomes more hydrated and pliable, leading to progressive softening of the substrate. Increased hydration can improve particle binding and cohesion, resulting in a better “bite” sensation, and can contribute to greater tenderness and juiciness (5, 120, 121). The second mechanism is mycelial structuring, in which the growing network of intertwined hyphae contributes directly to texture. As fungal growth progresses, given adequate permeability and interstitial space, the substrate is gradually overtaken and transitions from a loose or granular mixture (e.g., grains and legumes) into a biologically consolidated material. Hyphae extend, branch, and fuse, increasing their contact with substrate particles and establishing a cross-linked, fibrous scaffold throughout the substrate. The expanding network fills voids, wraps around substrate particles, and interlocks with neighboring hyphae, gradually forming the mycelial architecture that underpins matrix cohesion. Hyphal walls contribute to rigidity and elasticity, while the interconnected cords distribute mechanical stress and enhance adhesion. Collectively, these processes alter how the material fractures, compresses, and retains moisture, which creates a product with firmer, integrated, and more enjoyable textures (19, 21, 27). When mature and dense, this hyphal network can be modulated to create a “whole-cut” mycelium steak or meat-like texture where the grown matrix itself provides an anisotropic, meat-like bite. This is complemented by the liberation of *γ*-glutamyl peptides and glutathione which signal the sensation kokumi (“mouthfulness”), enhancing the perception of richness alongside umami (122–125). Thus, SSF can help make plant-rich staples more palatable while simultaneously increasing nutritive value via concentration and increased bioavailability.

For consumer-experience, framing seems to greatly influence consumer acceptance of fermentation biomass foods. Notably, contextual framing, especially anchored in the familiar, or that employs heightened emotional and sometimes sensory primes seems to be most influential (126). US consumers appear more receptive to novel-protein foods when they are framed around benefits (e.g., “clean,” “sustainable,” “high-quality protein”) than when labeled with process-focused terms such as “fungal” or “fermented,” which can trigger perceived “off” flavors, neophobia, or processing aversion (6, 127–129). By contrast, when these foods are positioned as flavorful, or protein-enhanced versions of familiar foods (e.g., high-protein noodles, breads, burger patties), adoption improves, including increased acceptance of tempeh in markets with less historical demand (130).

Allergen- and label-sensitive consumers often value short ingredient lists which is another advantage for SSF foods, as seen with successful market leaders like Quorn (131). In Western samples, tempeh often faces adoption barriers tied to cultural unfamiliarity and a fermented flavor profile with less enculturation, despite the strong nutritional profile (112, 126, 132). A synthesis of western consumer studies finds higher acceptance for alternatives that mimic conventional meat in texture and appearance, while tofu and tempeh tend to be perceived as niche or transitional products (110, 112, 132–135). Strategic benefit-forward framing—such as culinary versatility, environmental impact, and protein quality and digestibility—combined with sensory optimization (umami/kokumi chemistry, roasted volatiles) and familiar formats (patties, cutlets, sliced “whole-cut” mycelium) improves stated liking and purchase intent (6, 8, 15, 106, 136). Collectively, SSF foods, flours, and powders can be used to create minimally processed, clean-label platforms to boost protein, umami, and bioactives across familiar foods, aiding adoption (19, 44, 89, 120, 121, 137, 138, 139).

## As a strategy to improve diet quality and nutrition security

As discussed above, across cereals, legumes, and and by-products such as oilseed meals, filamentous fungi reduce anti-nutritional factors (e.g., phytate, sinapine, glucosinolates) and increase protein quality, digestibility, and micronutrient accessibility (3, 9, 28, 41, 115, 140, 141). Traditional examples such as tempeh and oncom illustrate that fungal proteases and peptidases liberate amino acids, and partially hydrolyze structural polysaccharides, which reduces structural rigidity and increases matrix deformability, improving digestibility (10, 14, 15, 66, 111, 123, 140, 142, 143). As a result, many SSF foods resemble meat when cooked, similar to widely consumed culinary mushrooms, while offering additional nutritional and sensory benefits (3, 15, 109, 142). SSF therefore can provide an abundant source of minimally processed, protein-dense, fiber-rich, low-saturated-fat foods that meet public-health guidance without sacrificing eating pleasure.

For nutrition-security, SSF upgrades abundant, affordable substrates with a range of nutrient densities, converting these into foods people choose for taste as well as health while reducing food waste. Traditional foods such as tempeh (*Rhizopus*-fermented soybeans) and oncom (*Neurospora*- or *Rhizopus*-fermented press cakes) have demonstrated for centuries that SSF can reliably extend the shelf-life of crops and side streams, enhancing global trade and minimizing waste. Compared to conversion of imperfect, surplus or side stream materials into other products, SSF can streamline operations (1, 2, 100, 144) and support immediate food applications with production cycles ranging from 36–48 h for *Rhizopus* to 10–21 days for *Pleurotus*. This means that production can be matched to demand, sited close to crop processors, and leveraged for rapid acceleration in production of quality protein sources. In contrast, common animal sources—fed the same underlying crops—require 2–24 months to reach market weight (14, 100). Plant protein sources take 90–120 days to harvest, but SSF can be grown on extant or rejected crops and side streams, which are typically low in protein quality pre-fermentation. Instead, SSF upgrades existing plant materials into higher-quality protein foods in a fraction of the time. This is also notable because many plant proteins, even high quality sources such as soy (PDCAAS = 0.92), remain limited in essential amino acid balance and digestibility, whereas SSF can raise PDCAAS values >0.96 through a combination of amino acid liberation, anti-nutrient reduction, and improved digestibility. SSF thereby enables rapid, demand responsive production of nutrient-dense protein foods faster than crop or animal foods could be produced, strengthening resilience during routine operations and supply disruptions (14, 100).

SSF also adds edible output per hectare and lowers delivered cost-per-nutrient. Unlike LSF systems, SSF uses the edible substrate as both growth matrix and food, avoiding expensive clarification and separation steps and large steam-in-place bioreactors. SSF tray and rack or tunnel systems using low-tech humidifiers and gentle aeration are sufficient (such as those for on-farm mushroom cultivation), and thus enable co-location with producers and processors for efficient side-stream valorization (1, 2, 144–146). Consider rapeseed (i.e., canola), where approximately 40% of the harvest produces oil and 60% remains as seed meal, or roughly 1.8 t/ha meal for every 3 t/ha grown. Typical concentration of protein in canola meal is about 38%, or 0.684 t protein/ha. The advent of *Pleurotus* increases the protein fraction by 11–18% (Table 1) while protein digestion inhibitors are reduced by 55–99% (28, 82). If assuming an average protein increase to 43.7% (15% relative) and total mass loss to respiration of 0–10%, this would result in a net increase in edible protein to between 0.708–0.787 t/ha. In other words, SSF would produce up to an additional 24–103 kg protein/ha. On the human scale, that incremental gain alone would supply up to 5.6 adult-years of protein per hectare, per year [based on international recommendation levels (147)]. This would also increase iron and zinc bioavailability from phytate removal and improve sensory attributes that may drive higher real-world adoption compared to the standard plant crops grown on the same land (9, 28, 41, 82).

SSF provides a pragmatic route to improving diet quality and food security by transforming low-cost crops and agricultural side streams into nutrient-dense, appealing foods. Its minimal processing requirements, short production cycles, and strong sensory performance align with public-health goals for protein, higher fiber, and lower saturated fat. SSF also advances U. N. sustainability priorities by strengthening circular-bioeconomy practices—including co-location and waste minimization—while avoiding ultra-processing and dependence on long, fragile supply chains (110, 116, 145, 146).

## Substrate selection strategies for fungal SSF

Substrates (Table 3) for SSF should provide carbohydrates, protein, nitrogen, and minerals, while minimizing factors that inhibit fungal growth (e.g., extreme pH, toxins) (3, 38). Common substrate categories include cereals, pseudocereals, legumes, oilseed cakes, omega fatty-acid-dense seeds, aquatic biomass (148–150), and bran-rich material from agricultural side streams (99). Effective substrate design blends materials to achieve an optimal carbon:nitrogen ratio and moisture level, while targeting desired nutritional outcomes. For example, a mixture of 70% cereal grain and 30% legume press cake can supply the needed carbon and nitrogen while yielding a balanced protein-fiber end product. Modifying substrate composition can significantly influence fungal performance and the resulting nutritional and sensory attributes (38).

**Table 3:** Food-grade substrate classes used for solid-state fermentation (SSF) with culinary fungi.

Substrate	Typical examples	Baseline nutrition	SSF Outcomes	Formulation notes	Applied examples
Whole cereal grains & pseudocereals	Wheat, rice, maize, barley, oats, millet; buckwheat, quinoa	High starch, moderate protein; bound phenolics & phytate common	Increase protein concentration and/or digestibility; Increase free phenolics/antioxidants; Decrease phytate; added umami/volatiles and other sensory gains via fermentation metabolites	Steam/gelatinize grains or hydrate to support colonization; adjust moisture (commonly ~60–65% wb); control particle size and aeration to modulate moisture; inoculate warm; mill post-SSF for high-protein flour	Whole-grain cereal tempeh fermentation reduced phytate (oats/barley; Rhizopus) (41); barley tempeh increased Fe absorption vs. boiled barley (9); Diverse cereal grains show increased phenolics/antioxidant under (93); more grain-fermentation phenolics effects (32). phenolics effects (32).
Legumes	Soybeans, chickpeas, lupin, black bean; legume flours	High protein (often 30–50% db), PUFA, protease inhibitors, lectins, phytates; beany notes (bitter phenolics)	Increase protein quality & digestibility (proteolysis);increased free amino acids; decrease in many anti-nutrients; EAA profile improved; increased phenolics and antioxidants including phenol pigments	Balance moisture and oxygen; consider dehulling/cooking to reduce flatulence factors and improve texture; co-blend with cereals when additional carbon is needed	Soybeans × Rhizopus oligosporus SSF (98). Black bean SSF with fungi increased antioxidative activity and phenolics (30). White lupin SSF reduced allergenic peptides (proteomics) (101).
Oilseed, meals and press cakes	Canola/rapeseed meal, cottonseed cake, sunflower, sesame, coconut; okara and other protein-rich byproducts	High protein (~30–50% db), variable lipids; constraints include sinapine/glucosinolates (Brassica) or gossypol (cottonseed); bitterness/astringency	Increased protein concentration and digestibility; reduced brassica anti-nutrients; detoxification of gossypol	Co-ferment with a cereal fraction when C: N is limiting; manage residual oil to avoid hydrophobic zones; verify food-grade sourcing (esp. cottonseed)	Canola meal × *Pleurotus ostreatus* SSF degraded sinapine and glucosinolates (28). Canola-meal upgrading (28). Cottonseed cake + Lathyrus pericarp x *P. ostreatus* increased β-glucans and decreased gossypol (29). Wheat grains and soybeans × *P. ostreatus* nutritional improvements (33).
Fiber- & bran-rich byproducts	Wheat/rice bran, corn fiber; fruit/veg pomace (apple, grape, pumpkin); spent brewers’ grains	High insoluble fiber, micronutrients; low protein; bound phenols	Increase extractable phenols and antioxidants; partial fiber depolymerization softening fiber matrix; increased moisture holding capacity	Use as a fraction (e.g., 10–40%) blended into cereal/legume/oilseed bases to maintain growth; fine grinding improves uniform colonization; ensure low contaminant load and safe water activity	Wheat SSF × Rhizopus oryzae increased production of phenols (85). SSF as platform to produce antioxidant polysaccharides (66); cereal-grain antioxidants via SSF across fungi (93).
Omega-rich seeds & lipid sources	Flaxseed, chia seed; defatted seed meals; oil press cakes	ALA/PUFA, lignans	Can improve ω-3 content and ω6:ω3 ratio in the composite when lipid fraction is blended into a carbohydrate-supporting matrix; retains PUFA while fungus consumes carbs	Pre-mix finely; excess free oil can inhibit if pooled, limit oil to avoid hydrophobic zones; combine with cereal/legume	Grass pea tempeh + flaxseed oil-cake improved nutritional value (including lipid profile) (99).
Microalgae and aquatic biomass	*Chlorella vulgaris* (typically blended with cereals); Arthrospira/Spirulina (often with stabilization)	High protein and pigments; bound phenols; sensory off-notes or fishy odors; benefits from a carbohydrate carrier	Increased water retention and gellification/emulsification; broader micronutrient enhancements	Use as minor fraction (e.g., 5–30%) in cereal/legume composite; pre-treatments help support integration	*P. ostreatus* SSF on oat and Chlorella + oat improved protein solubility (149). Arthrospira SSF (148). SSF of Sargassum macroalgae with Aspergillus oryzae (150)

Above include observed changes in SSF foods from baseline composition (e.g., protein concentration, phytate/glucosinolate reduction, antioxidant/umami increases), and key formulation levers with representative applied examples. Substrate class boundaries overlap in practice; most edible SSF products are blended matrices designed to optimize C: N ratio, moisture/water activity, and aeration.

## Growth conditions for optimizing SSF

Numerous factors can be manipulated to impact the microenvironment around hyphae, influencing the physiochemistry of the mycelium, and the time to maturity, including: substrate moisture, substrate nutrients, water activity (aw), temperature (and heat removal), particle size, bed depth, and aeration, agitation, inoculum load and distribution, and ambient relative humidity. Fungal growth typically requires high aw (appx. 0.95–0.99), but not as free water; excess and/or localized moisture collapses inter-particle pores, choking oxygen transfer and limiting enzymatic activity and biomass formation (2, 151). Heterogeneity in growth bed construction can create moisture and temperature gradients that alter colonization rates and metabolite production. Gentle forced aeration (e.g., systematic agitation or vibration) helps distribute oxygen and moisture, and when paired with bed design, can support robust growth and reproducible yield characteristics (1, 2, 105).

Optimal conditions for growth include temperatures between 24 and 30 °C, with relative humidity (RH) between 85 and 90% to minimize evaporative losses, in shallow beds that facilitate easy heat removal. When temperatures exceed 35 °C, common species (e.g., *Pleurotus*) experience slowed mycelial growth and thermal hotspots that can result in quality loss, reduced yields, or stunting, suggesting that forced-air systems should provide sufficient O₂ while simultaneously delivering gentle cooling in trays or packed beds. Growth conditions impact time-to-maturity, with tempeh-style *Rhizopus* systems typically reach a cohesive, sliceable cake in as little as 24–48 h at 30–35 °C and 85–90% RH, whereas *Pleurotus* tray cultures require several days to weeks, influenced by bed depth, inoculum density, and heat management (50, 152).

Substrate engineering also strongly influences mycelia growth. Particle size and distribution can create interparticle air gaps that promote gas diffusion, providing ample surface for hyphal attachment and penetration. In contrast, overly fine particles increase bed resistance and restrict oxygen transfer, and excessively coarse fractions hinder colonization uniformity and may prevent fungal access to nutrient dense starch stores beyond hard cell wall barriers. Mild pre-treatments to the substrate (hydration, gelatinization for starch-rich cereals, or mild enzymatic conditioning) can accelerate colonization. Buffering, or initial acidification of the substrate (e.g., to pH 4.5, as in tempeh manufacture) provides an effective microbial hurdle, suppressing spoilage bacteria during early stages of fermentation while remaining permissive to fungal germination and early hyphal growth. Once the fungus established, pH tends to drift toward neutral pH (6.5–7) as organic acids are metabolized (153).

Circadian and photic cues are increasingly recognized as major regulators of fungal metabolism and development, yet most food-grade SSF protocols employ steady, constant setpoints (entrainment of darkness, temperature and RH). *Neurospora* is the canonical fungal strain for the circadian clock model where endogenous transcription–translation feedback loops have been seen to entrain robustly to both light/dark and cool/warm temperature cycles with significant resultant effects on primary and secondary metabolism, conidiation, and stress responses (154–156). These principles generalize across fungi with light acting through conserved photoreceptors to reprogram metabolism and development; thus, timed light/temperature cues can modulate metabolic flux—i.e., the rate of carbon passage—through glycolysis and the pentose-phosphate pathway, alter redox balance, and shift volatile and pigment profiles (157, 158). Although basidiomycetes used in foods are less clock-mapped than *Neurospora*, *Pleurotus* spp. clearly perceive blue light via White Collar-1/2 homologs, and blue-light exposure modulates primordia formation and central carbon metabolism, and are responsive during vegetative growth stages in SSF (159, 160). SSF process design could thus improve yields and sensory outcomes by moving beyond static setpoints and toward programmable circadian-optimized regimes to allow synchronized hyphal growth and enzyme secretion, reduce heat-of-metabolism bottlenecks, and potentially increase desirable flavors and pigments.

## Food system benefits: sustainability and food security

SSF offers value-added endpoints for commodity crops and agricultural side streams alike. Low-value side streams, esthetically imperfect main crops, and edible by products are underutilized materials and a significant valorization opportunity to bolster rural economies, and improve nutrition security. Valorization allows the same agricultural inputs (e.g., land, water, fertilizer) used to grow main crops to feed more people, improving the resource efficiency of the food system (3, 11, 102, 161–163). Many crops produce a large fraction of inedible or underutilized residues (e.g., nut hulls, cereal brans, seed cakes, fruit pomace) that are typically relegated to edible low-value animal feed, or inedible low-value streams like packing and fillers, when not fully lost as farm waste (29, 75, 164). Utilization for SSF thereby directly addresses U. N. Sustainable Development Goal for Zero Hunger and Responsible Consumption/Production, by increasing the food supply without clearing additional land for cultivation and minimizing waste (3, 11). For one example, in global peanut production, hulls are >20% of the yield and are mostly used as feed or processed into packaging materials (164). Through SSF, peanut hulls can feed flavorful nutrient dense mycelium, boosting the total human-edible output per hectare, increasing farm diversification, and contributing to food security in peanut-growing regions.

SSF can further support food security through nimble and strategic deployment owing to its low resource requirements and utilization of simple, modular infrastructure (165). SSF cultures can be distributed in compact, lightweight, lyophilized formats that are shelf-stable and easily rehydrated, allowing diverse local substrates to be inoculated with minimal inputs (166, 167). This enables rapid establishment of food-production systems in resource-constrained or decentralized settings, a capability especially valuable following natural or political disasters—now recognized as an urgent global food-security priority (168, 169). This allows hyperlocal activation of regionally available foodstuffs, or waste and by-product streams, with a capacity to produce nutritionally enhanced protein foods quicker than legacy agricultural systems. Temporal decoupling and local resource valorization position SSF as a promising solution for food security in constrained environments, including long-duration space missions (170, 171).

Fungal fermentation has a relatively low environmental footprint compared to many traditional protein sources. Mushroom and mycelium emits fewer greenhouse gases and uses less water and land than raising livestock. For instance, Quorn’s Carbon Trust–certified footprint analysis results report a farm-to-factory-gate carbon footprint for Quorn mycoprotein (*Fusarium venenatum*) of 0.7 kg CO2 per kg mycoprotein, reporting a footprint that is 55 × lower for carbon, 13.5 × lower for water, and 5.5 × lower for land use than beef (172). Additionally, mushrooms and mycelium can be grown indoors, minimizing competition with field agriculture for land, extending production throughout the year and multiple regions, while buffering against seasonal disruptions. Fungi also exhibit high substrate-to-biomass conversion efficiency because they expend no energy on locomotion or thermoregulation, unlike livestock. In optimized systems of edible SSF such as tempeh, fungal biomass is predicted to comprise approximately 5.9% of the final product on a dry-weight basis, whereas harvested mycelial-mat systems, such as those using *Pleurotus* report yields of over 12% accrual above starting substrate mass (2, 173, 174). These biomass gains are not all substrate conversion, as prolonged SSF with some fungi can incur dry-matter losses up to 30.9%. Substrate utilization and biomass accrual are tunable by growth conditions and strain, with commercial endpoints aiming for products with closer to 50% fungi and 50% plant-based remaining substrates (175).

## SSF for farm economies

SSF offers downstream and on-farm revenue diversification for farmers and local producers. The increasing global consumption of animal protein has resulted in larger proportions of arable land used to grow crops for animal feed, rather than human food. Over half of habitable land is currently used for agriculture, with over 80% estimated to be used for livestock between raising, grazing, and feedstock (176, 177). Over recent decades, approximately 36% of global crop calories grown are currently used for animal feed globally, with over a third of croplands devoted to feed crops. In the US, most corn in a high-concentration corn growing region, goes to livestock rather than human foods (176, 178, 179). By creating additional human demand for crops and agricultural byproducts, SSF can increase revenue from the same raw materials for rural economies. Rather than selling these materials solely as animal feed, farmers could supply them to fermentation manufacturers or implement on-farm fermentation to reduce waste and lower transportation costs between process steps. Studies also show that upcycled side streams command higher prices when repurposed for human food than when sold as low-value feed or fuel (29, 75, 164). Further, production of on-farm fungi dovetails with many national US and international initiatives for rural development. For example, oyster mushroom cultivation is already promoted by the Food and Agriculture Organization (FAO) of the UN and the United States Department of Agriculture for farm diversification as a low-investment, high-nutrition crop that farmers or cooperatives can grow on wastes (180–182). SSF extends this by expanding the range of substrates for growth and resulting end products, which can help combat rural malnutrition and command premium prices from staple commodity crops, giving farmers access to higher-value markets.

## Commercial developments and emerging mycelium-based foods

Mycoprotein products have been on the market for several decades, but recent interest has increased the number of enterprises leveraging fermentation technology for novel foods. Fungal foods now span industrial mycoprotein, traditional SSF staples (e.g., tempeh), whole-cut mycelium meats, and protein fortified flours and flavor dry ingredients. Quorn™ pioneered large-scale mycoprotein (submerged *Fusarium venenatum*) and proved mainstream appeal of its high protein and higher fiber chicken alternatives (172). Southeast Asian classics like tempeh (soybeans + *Rhizopus*) and oncom (peanut and/or soy + *Neurospora*) demonstrate SSF’s ability to boost digestibility, vitamins, and savory flavor. Newer platforms on the market have shown thick *Pleurotus* mycelium “slabs” like the MyForest/Atlast sliceable bacon; *Rhiza* produced by Better Meat Co. as a fast-growing filamentous fungus on low-value plant potato waste; *Neurospora* strains producing cutlet-style products like Meati, and the *Koji* deli meat formats of Prime Roots that produce fibrous mycelia in submerged tanks or trays (172). Aside from meat analogs, mycelium is increasingly used as a functional processing step—debittering and improving plant proteins (e.g., shiitake-treated pea and rice), fortifying flour, and to generate enzyme- or antioxidant-rich ingredients for breads, gravies and other foods—highlighting the versatile, clean-label potential (66, 183).

As consumers grow more conscious of health and sustainability, fungi-derived functional foods are poised to capture a significant share of the protein market. For example, Quorn notes that their increased fiber ‘may help regulate blood cholesterol levels’, and Meati emphasizes that their mycelium is packed with ‘protein, vitamin B12, and minerals, while being low in fat’. Alternatively, high-protein SSF flour can be used to fortify breads, tortillas, and snacks, or dried and micronized into natural umami-rich seasoning powders (19, 44). Wheat bread with *Pleurotus ostreatus* amendments showed increased protein and antioxidants while retaining good loaf and crumb texture (121); similar approaches to functional enhancements in bread with *P. eryngii* increased nutrient density and observed acceptable liking scores from tasters (137), as with *Agaricus* and *P. sajor-caju* fortified breads (138, 139) cookies, and snacks (120).

Compared with LSF, SSF is underutilized because its core engineering and control problems are harder to solve at scale. Heterogeneous moist beds impede heat-mass transfer, creating local hot spots, moisture/aw and O₂/CO₂ gradients that reduce reproducibility and complicate scale-up—issues largely tamed in stirred, well-mixed LSF bioreactors (1, 2, 4, 105). SSF also lacks mature on-line instrumentation, with key conditions such as moisture, metabolic rate, and endpoint prediction often inferred indirectly (respirometry, NIR) rather than through robust probes common to LSF (1, 100). Feedstock variability further raises transaction costs—agri-byproducts require food-grade presorting, particle-size/voidage control, and thermal pre-treatments for safe, uniform colonization—whereas LSF typically uses standardized liquid media (144–146). Microbiological risk management for SSF depends on bed hygiene, defined GRAS starter cultures (Generally Regarded as Safe, or comparable determinations in regions outside of the US), and early acidification and heat to suppress competitive biological contaminants and mycotoxin risks, whereas LSF leverages closed vessels and antibiotic-free asepsis (14, 16, 184, 185). Finally, capital ecosystems, regulatory familiarity, and workforce experience are deeper for LSF (ubiquitous tanks, sensors, etc.), so investment and know-how naturally pool there despite SSF’s potential low-capital expense (CAPEX), low-utilities advantages (2, 144). SSF and LSF are thus best viewed as complementary platforms: LSF excels at producing homogeneous biomass and more purified ingredients under tightly controlled, high-water conditions, whereas SSF enables low-water transformation of solid food matrices that can increase intake of both the substrate and fungal biomass as a minimally processed composite food, with distinct constraints and advantages.

SSF-derived mycelium is a promising scaffold for anchorage-dependent cell cultures in the production of another emerging food technology—cell-cultivated meat (sometimes referred to as in-vitro, or lab-grown meat) (186). For example, *Pleurotus ostreatus* mycelium has been shown to support bovine satellite cell adhesion and proliferation, providing a structurally edible matrix with tunable porosity and mechanical integrity (187). Additionally, the use of *Ganoderma lucidum* mycelium in such hybrid constructs, showed enhanced cell viability and nutrient diffusion compared to more common collagen-based scaffolds. These platforms both reduce reliance on animal-sourced matrices and enable sensory and nutritional optimization.

Mycelium scaffolds produced by SSF can be nutritionally crafted with substrate design and process control. For example, co-fermenting legumes and cereals with flaxseed press-cake or other *ω*-3–rich inputs shifts the lipid profile to be higher in *α*-linolenic acid with favorable ω-6:ω-3 ratios, while retaining fungal protein and dietary fiber (99). Beyond composition, the intertwined anisotropic structures can function as a fungal connective-tissue armature that can be further shaped by moisture, particle size, voidage, and growth time for desired composition. This approach can offer sensory and structural enhancements when paired with pea-protein systems for meat analogs (28).

Meat-like coloration can also be enhanced with pigments from fungal fermentation. For example, *Neurospora* generates carotenoids (e.g., neurosporaxanthin) and *Monascus* red pigments provide heat-stable red/pink hues suitable for raw and cooked analogs, offering cleaner-label routes to natural and edible colorants with nutritional value (188–190). This approach potentially has direct applications within cell-cultivated meat and fish technologies, and represents a convergence of bioprocessing and tissue engineering. The ability to combine natural and familiar mushroom-derived processes into plant-based and other novel protein foods, can help meet consumer needs, public health nutritional and gastronomic goals (191).

## Food safety and regulatory considerations

SSF with edible fungi is safe when producers pair pure, food-grade starter cultures with aseptic, well-controlled processes to suppress contaminants, prevent toxins, manage allergens, and comply with applicable regulation. Fast-colonizing strains give the inoculum a competitive edge able to outcompete adventitious agents and later spoilage organisms. Together, with substrate pasteurization or sterilization and pre-acidification, the pathogenic risk is reduced. Careful development of Good Manufacturing Procedures (GMP) and Hazard Analysis and Critical Control Point (HACCP) frameworks can also specify sterilization, clean inoculation, and end-point moisture as critical controls among others. Using defined monocultures improves reproducibility and safety (5, 14, 192).

Strain choice and other process conditions can also mitigate risk. Edible species such as *Pleurotus, Agaricus, Lentinula*, and tempeh’s *Rhizopus oligosporus* are used in food fermentation without problematic mycotoxin production. For example, domesticated *Aspergillus oryzae* (koji) used for centuries in traditional foods and in modern commercial applications (e.g., Prime Roots deli meats) has a long history of safe food use. Comparative genomic studies showed that the domestication of *koji* derived from atoxigenic lineages (193, 194). Aside from the in-field use of atoxigenic *Aspergillus flavus* strains as biocontrol pre-harvest, post-harvest biodegradation/biotransformation approaches include fungal- and enzyme-mediated transformation of aflatoxins under controlled conditions, including reports using *Trichoderma reesei* (16, 193, 195–198). Laccases/peroxidases produces from many strains have also been demonstrated in controlled LSF fermentation to oxidize aflatoxins (199). Companies (e.g., MyForest/Atlast) have argued that mycelium is “substantially similar” to fruiting bodies of the same mushrooms, supporting low-novelty safety claims. Producers have also gone to great lengths to demonstrate methodological and end point safety, including to verify absence of mycotoxins in products such as *Fusarium mycoprotein* (Quorn) as part of GRAS submissions (175, 200–203). At industrial scale, safety programs track pH, moisture, and temperature combined with kill steps, protective packaging, and traceable food-grade inputs. Regulatory pathways vary by region: in the U. S., many edible fungi qualify via GRAS or food-additive petitions (e.g., Fusarium venenatum mycoprotein; GRN 945), and oyster-mushroom mycelium has been the subject of a recent GRAS notice by Mushlabs [GRN 1152, submitted but FDA ceased review in 2024 (204)]; other firms (e.g., Better Meat Co.’s *Rhiza* mycoprotein) have received “no questions” letters (205). In the EU, several mycoproteins entered via Novel Food authorization. Ongoing calls for harmonized standards tailored to mycelium protein emphasize process control, toxin/allergen risk management, and accurate labeling, reflecting regulators’ receptivity when dossiers are robust (203, 205, 206).

## Discussion

SSF using culinary mushroom fungal strains can be a practical bridge between traditional fermentation and next-generation sustainable foods. By working directly on solid substrates, SSF—unlike LSF—can transform intact agricultural crops into minimally processed, flavor-forward, high-protein foods, enhancing bioavailability and palatability without reliance on isolates or ultra-processing. For more sustainable food systems, SSF can help to reduce food waste and increase food supply efficiency by valorizing commodity crops and side streams, producing high-quality protein with a low environmental footprint, creating economic opportunities for agriculturalists and food manufacturers alike (172).

Results in compositional shifts and tunable factors on outcomes were generally directionally consistent, but magnitudes and functional implications varied substantially across substrates, fungal strains, process conditions, and outcome measures. Some studies show reduced allergenic peptides or altered allergen bands, these may not all consistently translate into large reductions in IgE binding or proven clinical benefit. Similarly, for nutritional endpoints, many studies reported higher protein percentage, but sometimes this may reflect a shift in relative concentration from dry-matter loss, versus absolute protein biomass increases. For consumer acceptance, optimized SSF products often received higher preference ratings than traditional formats like tempeh/oncom, but these products may still face rejection from consumer groups who are unfamiliar with these foods. While these are not contradictory scientifically, it does indicate that results are format- and context-dependent, and multifactorial explorations that can more directly cross-compare are needed.

Demand for protein foods is expected to outpace supply by as soon as 2050, creating an important and timely need for innovations that not only deliver more protein foods but that can effectively meet the acceptance needed to serve that demand (17, 207). Current gaps in understanding include the generalizable, direct, mediated, and synergistic quantitative links between (i) substrate precursors and enzyme portfolios, (ii) process variables and variable schema (moisture, particle size, temperature, aeration, mono- vs. co-culture), (iii) methods for environmental and stress provocation of chemical drivers of taste and aroma, (iv) perceived sensory outcomes and resultant substitution and adoption, and (v) and consequential techno-economic outcomes when optimizing for any of the above. Resolving these unknowns calls for factorial SSF studies that integrate targeted functional analytics, comparisons of pre/post cooking methods, tied to predictive process–product-adoption maps. Producing a validated design framework that maps these interdependencies would de-risk scale-up, guide co-culture selection, and accelerate minimally processed SSF foods toward nutrition security and commercialization. With optimized processes, SSF can be scaled to produce delicious, health-promoting foods that are cost-effective to manufacture and affordable for consumers, while increasing yield and shortening time-to-harvest—benefits that strengthen both farm economies and nutrition security. Continued optimization can also support the development of improved edible fungal strains that grow more rapidly, generate specific health-promoting compounds, and more efficiently utilize diverse inputs, enhancing nutritional profiles and expanding waste-valorization opportunities.

## Limitations of SSF and SSF research—future directions

Current SSF research remains methodologically limited by small-scale, strain-specific studies that use heterogeneous substrates, process conditions, and outcome measures, making cross-study comparison difficult. Many studies report compositional change but do not pair those data with all of the necessary components summarized above including analytically verified bioavailability, trained sensory evaluation, consumer acceptance, or shelf-life and safety endpoints in the same system. Few studies have characterized the total digestible or bioavailable protein of mycelium-based foods produced through edible-substrate SSF, or calculated their PDCAAS. Reporting of key engineering variables is also inconsistent, including water activity, moisture distribution, bed depth, inoculum load, particle size, aeration, and heat removal, even though these strongly influence colonization, metabolite formation, and reproducibility. As a result, the field still lacks generalizable multifactorial rules and validated factorial designs, standardized controls, or technoeconomic endpoints that would allow robust comparison across food-grade SSF formats.

Industrial translation presents additional challenges. Relative to LSF, SSF is harder to standardize because moist beds create localized gradients in temperature, oxygen, carbon dioxide, and moisture that increase contamination risk and complicate process control. Feedstock variability also raises requirements for preparation and hygienic handling. Practical scale-up will therefore depend on sourcing, hazard controls, and better real-time monitoring, and as complex novel foods would be best supported by clearer regulatory pathways from supply side to market. Although SSF may offer lower capital and utility demands than stirred-tank systems, the economic feasibility is not yet well resolved and some advantages may be offset by labor intensity, batch variability, and the need to innovate novel commercial equipment. Future work should therefore prioritize factorial studies that link process variables to nutritional, sensory, safety, and yield outcomes, together with standardized techno-economic and life-cycle assessments to clarify where SSF is most competitive and scalable.

## Conclusion

SSF is a promising approach to pressing food challenges. It provides a route to improve human nutrition by merging the strengths of plant foods and fungal biotechnology, yielding products that can help diversify protein sources and mitigate malnutrition while providing consumers whole foods with ingredients they will recognize and public health officials can endorse. SSF can help sustainably produce more minimally processed plant foods and bolster circular economies. While challenges in optimization, scaling and consumer education remain, the progress to date signals that fungal fermentation can indeed move from the realm of niche research and traditional practice into the mainstream of global food supply. With continued interdisciplinary effort, mushroom mycelium foods could emerge as a staple contributor to high-protein, functional foods that support human and planetary health, while enhancing rural economies in the years ahead.
